# RNAi-Mediated Specific Gene Silencing as a Tool for the Discovery of New Drug Targets in *Giardia lamblia*; Evaluation Using the NADH Oxidase Gene

**DOI:** 10.3390/genes8110303

**Published:** 2017-11-03

**Authors:** Jaime Marcial-Quino, Saúl Gómez-Manzo, Francisco Fierro, Yadira Rufino-González, Daniel Ortega-Cuellar, Edgar Sierra-Palacios, America Vanoye-Carlo, Abigail González-Valdez, Angélica Torres-Arroyo, Jesús Oria-Hernández, Horacio Reyes-Vivas

**Affiliations:** 1 CONACYT-Instituto Nacional de Pediatría, Secretaría de Salud, Ciudad de México 04530, Mexico; 2 Laboratorio de Bioquímica Genética, Instituto Nacional de Pediatría, Secretaría de Salud, Ciudad de México 04530, Mexico; saulmanzo@ciencias.unam.mx (S.G.-M.); angelica.tarroyo@gmail.com (A.T.-A.); jesus.oria.inp@gmail.com (J.O.-H.); 3 Departamento de Biotecnología, Universidad Autónoma Metropolitana, Iztapalapa 09340, Mexico; degfff@yahoo.com; 4 Laboratorio de Parasitología Experimental, Instituto Nacional de Pediatría, Secretaría de Salud, Ciudad de México 04530, Mexico; yadirg@gmail.com; 5 Laboratorio de Nutrición Experimental, Instituto Nacional de Pediatría, Secretaría de Salud, Ciudad de México 04530, Mexico; dortegadan@gmail.com; 6 Colegio de Ciencias y Humanidades, Plantel Casa Libertad, Universidad Autónoma de la Ciudad de México, Ciudad de México 09620, Mexico; edgar.sierra@uacm.edu.mx; 7 Laboratorio de Neurociencias, Instituto Nacional de Pediatría, Secretaría de Salud, Ciudad de México 04530, Mexico; america_vc@yahoo.com.mx; 8 Departamento de Biología Molecular y Biotecnología, Instituto de Investigaciones Biomédicas, Universidad Nacional Autónoma de México, Ciudad de México 04530, Mexico; abigaila@correo.biomedicas.unam.mx

**Keywords:** *Giardia*, NADH oxidase, drug targets, RNAi, siRNA, stem-loop RT-qPCR

## Abstract

The microaerophilic protozoan *Giardia lamblia* is the agent causing giardiasis, an intestinal parasitosis of worldwide distribution. Different pharmacotherapies have been employed against giardiasis; however, side effects in the host and reports of drug resistant strains generate the need to develop new strategies that identify novel biological targets for drug design. To support this requirement, we have designed and evaluated a vector containing a cassette for the synthesis of double-stranded RNA (dsRNA), which can silence expression of a target gene through the RNA interference (RNAi) pathway. Small silencing RNAs were detected and quantified in transformants expressing dsRNA by a stem-loop RT-qPCR approach. The results showed that, in transformants expressing dsRNA of 100–200 base pairs, the level of *NADHox* mRNA was reduced by around 30%, concomitant with a decrease in enzyme activity and a reduction in the number of trophozoites with respect to the wild type strain, indicating that NADHox is indeed an important enzyme for *Giardia* viability. These results suggest that it is possible to induce the *G. lamblia* RNAi machinery for attenuating the expression of genes encoding proteins of interest. We propose that our silencing strategy can be used to identify new potential drug targets, knocking down genes encoding different structural proteins and enzymes from a wide variety of metabolic pathways.

## 1. Introduction

*Giardia lamblia* is a unicellular flagellate protozoan causing giardiasis, which is catalogued as a global public health problem. Children and immuno-compromised patients are the main affected groups. This intestinal parasite has worldwide distribution, affecting humans, vertebrate and invertebrate groups [1,2]. Patients with giardiasis symptoms show different clinical manifestations, which range from diarrhea and vomiting to malabsorption syndrome and failure to thrive due to intestinal damage [2]. Compounds derived from benzimidazoles and nitroimidazoles have been used in pharmacotherapy against giardiasis [3]; however, side effects in the host and the existence of resistant strains to these drugs have been reported [3,4]. To overcome these problems, several groups have attempted different strategies to find new antigiardiasic compounds [3,5,6].

To deal with this challenge, molecular biology approaches can be used aiming to specifically attenuate expression of a gene encoding a putative target protein. In this context, the fact that *Giardia* trophozoites contain two nuclei, and the likelihood that such parasites exhibit polyploidy [7], actually makes gene knockout infeasible [8,9]. As an alternative, RNA interference (RNAi), a mechanism working through small RNA molecules to block translation and degrade specific mRNAs [10,11], may be a useful tool to selectively attenuate gene expression; this method has been successfully used for interfering with gene expression in several parasites [11,12].

There are few studies employing RNAi in *Giardia* [11,13,14,15]. Several elements of the RNAi silencing pathway have been identified in *Giardia*, making conceivable its existence and therefore its potential usefulness to perform specific gene silencing. Analysis of the *Giardia* genome has revealed the presence of one gene copy each of the Dicer (*Dcr*), Argonaute (*Ago*) and RNA-dependent RNA polymerase (RdRp) [13,16,17]. Although Drosha and Exportin 5 homologs are absent in this parasite, there exists the possibility of another Drosha-independent pathway involved in the processing of small RNAs [16,18]. The 3D structure of Dcr was solved, showing that it contains the Piwi/Argonaute/Zwille (PAZ) domain, which participates in the synthesis of small interfering RNA (siRNA) of 21–28 nucleotides [19,20,21]. *Giardia* Dcr (GlDcr) was proven to be functional in a *Schizosaccharomyces pombe* Dicer-deleted mutant [20]. Saraiya and Wang described that GlDcr is involved in the synthesis of microRNAs (miRNAs) derived from small nucleolar RNA (snoRNA), and that Ago of Giardia (GlAgo) participates in the miRNA-mediated silencing of the expression of proteins whose mRNA contain target sites for miRNA [16]. Recently, Guo et al. proposed that the variant-specific surface protein (*VSP*) genes are transcribed simultaneously to sense and antisense strands, resulting in double-stranded RNAs (dsRNA) which are then degraded by a Dicer endonuclease, and that this regulation also involves antisense VSP transcription carried out by RdRP [15]. These results confirm that *G*. *lamblia* possesses an RNAi-type mechanism of gene silencing with enzymes that generate small RNAs [11,13,14,16,22]. In this article we report the construction of a vector designed to drive the synthesis of dsRNA for gene silencing through the RNAi pathway, and its ability to knock-down expression of a possible target protein for drug design.

The NADH oxidase (NADHox; E.C.1.6.99.3) from *G. lamblia* (GenBank id: XP_001707974.1) was selected for this purpose. NADHox catalyzes the reduction of O_2_ to H_2_O via a free intermediate-electron transporter mechanism [23]. This enzyme depletes oxygen from cells to protect sensitive enzymes from oxidation, and it is expected to be critical because *Giardia* does not possess the conventional enzymes for detoxifying reactive oxygen species (ROS), such as glutathione reductase, peroxidase, catalase and superoxide dismutase [23,24].

Our results show that the constructed vector, containing two opposite-oriented promoters for dsRNA synthesis, is effective for silencing expression of *NADHox*, reducing the amount of its mRNA in the transformants, and resulting in a decrease in enzyme activity as well as cell growth. We confirmed the presence of siRNA molecules derived from the dsRNAs synthesized by sense and antisense transcription of the inserted *NADHox* gene fragments. This strategy makes it possible to routinely evaluate candidate proteins as potential drug targets in *G. lamblia*.

## 2. Materials and Methods

### 2.1. Primers Design

All primers used in this study are described in Table 1. The sequences for primer design were obtained from GenBank and from the GiardiaDB: The Giardia Genomics Resource [25]. The primer pairs were designed from DNA and mRNA sequences for promoters and genes, using the website Primer3 Input [26]. All primers were validated for their specificity by sequencing, and the primers used for reverse transcription quantitative PCR (RT-qPCR) were tested using melt curves and calculating amplification efficiency as previously reported by Marcial et al. [27].

### 2.2. Giardia Genomic DNA Extraction and Amplification by PCR

Total genomic DNA was extracted from *G*. *lamblia* using the DNAzol reagent (Invitrogen, Carlsbad, CA, USA) as described by the manufacturer. This extraction method was also used in the transformant *Giardia* strains to determine the integration of the vectors in their genomes. The extracted total DNA was used as a template to amplify promoter regions and the *NADHox* gene fragments. The promoter regions of glutamate dehydrogenase (*gdh*) (GenBank id: AEM66245.1) and *δ*-giardin (*δ-gia*) (GenBank id: AF331827.1) were amplified by PCR using the primers specified in Table 1. *Vent* polymerase (New England BioLabs, Ipswich, MA, USA) was used for all PCR amplifications and conditions were as follows: One cycle of 4 min at 94 °C; 30 cycles of 30 s at 94 °C, 30 s at 60 °C, 60 s at 72 °C; and one final cycle of 7 min at 72 °C.

### 2.3. Construction of Vectors pTubGdh-RNAi and pTubGdh_eGFP-RNAi

The vectors for the synthesis of dsRNA in *Giardia* were constructed as follows. We used a fragment of the plasmid pTub_H7_HA_PAC (a kind gift from Dr. Hugo Lujan, Universidad Católica de Córdoba, Argentina) [13,14], to isolate the promoter region of α2-Tubulin (*α2-Tub*) from *G*. *lamblia* and the puromycin N-acetyl-transferase gene (*pac*), which confers resistance to this antibiotic, transcribed from the *γ*-Giardin promoter region (*γ-Gia*) (GenBank id:X55287.1). After excising the H7 *VSP* gene from pTub_H7_HA_PAC by digestion with the enzymes NcoI and EcoRV and religation, we named the resulting vector pTub_HA_PAC.

**Figure 1:**
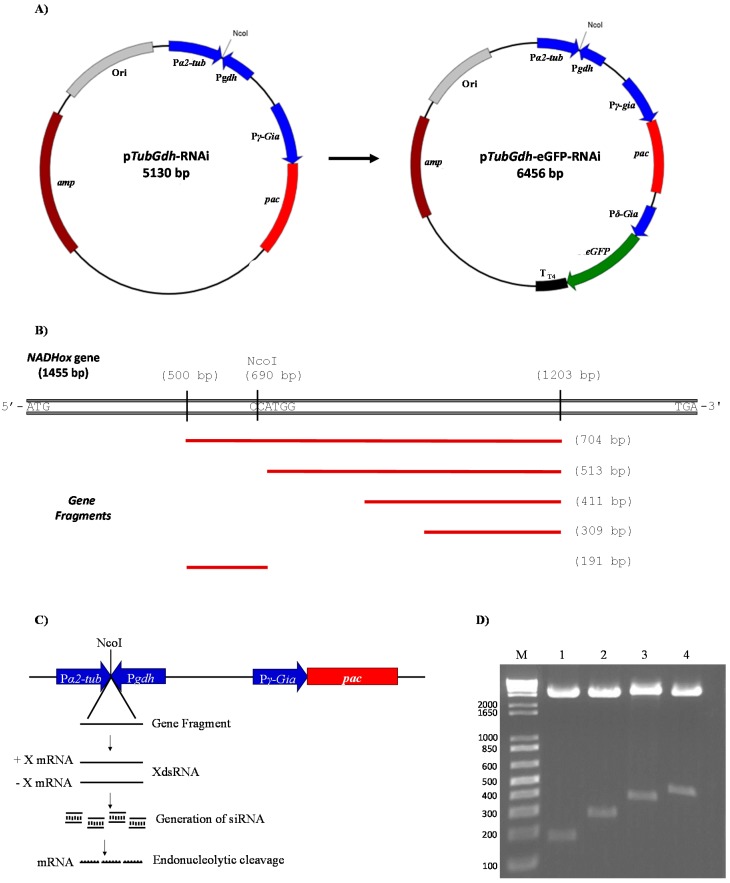
Vectors and strategy to generate double-stranded RNAs (dsRNAs) from the *NADHox* gene. (**A**) Map of the p*TubGdh*-RNAi and p*TubGdh*_eGFP-RNAi vectors, showing the position of the *α2-Tub*::*gdh* cassette with the *Nco*I cloning site. The *pac* gene, conferring resistance to puromycin, was used as selective marker for transformation. (**B**) Position of the fragments used for gene silencing of the *NADHox* gene. (**C**) Close-up of the *α2*-*Tub*::*gdh* cassette, showing the expected outcome after insertion of the *NADHox* gene fragments at the *Nco*I restriction site, with the generation of dsRNA from the corresponding fragment and subsequent silencing of *NADHox* expression through the RNAi pathway. (**D**) The insertion of the *NADHox* gene fragments into the vectors was confirmed by digestion with *Nco*I and electrophoresis; lanes 1–4 show the released fragments of approximately 200, 300, 400 and 500 base pair (bp) from vector p*TubGdh*_eGFP-RNAi, respectively, and lane M is the molecular weight marker (1 Kb Ladder, Invitrogen).

Next, the previously amplified *gdh* promoter was digested with EcoRV and NcoI, and ligated to the EcoRV + NcoI-digested plasmid pTub_HA_PAC. Hence, the NcoI site of the *gdh* promoter is linked to the 245 base pair (bp) region that belongs to the 3′-end of the *α2-Tub* promoter, whereas the EcoRV site of the *gdh* promoter is connected with the 190 bp region belonging to the 5′-end of the *γ-Gia* promoter. The construction was named p*TubGdh*-RNAi (Figure 1A). The construction of vector p*TubGdh*_eGFP-RNAi involved the insertion of the green fluorescent protein gene (*eGFP*) cassette into p*TubGdh*-RNAi using the restriction site SacI. The *eGFP* cassette contains the *eGFP* gene (GenBank id: CAD97424.1) transcribed from the *δ*-*Gia* (GenBank id: AF331827.1) promoter and with the terminator T_T4_ from bacteriophage T4 (Figure 1A).

Both vectors contain the promoters *α2-Tub* and *gdh* positioned facing each other and separated by a NcoI restriction site. This region was named *α2-Tub*::*gdh* cassette; the NcoI restriction site was used to insert fragments of different sizes from the *NADHox* gene for the synthesis of dsRNA. The *NADHox* gene fragments (of approximately 200, 300, 400 and 500 bp in size) were amplified by PCR with primers containing NcoI sites at the 5′-end (Table 1), then digested with NcoI and ligated to the vector. All regions amplified from the *NADHox* gene are shown in Figure 1B. The vectors containing the different fragments were named p*TubGdh*-RNAi_200_ through p*TubGdh*-RNAi_500_, and p*TubGdh*_eGFP-RNAi_200_ through p*TubGdh*_eGFP-RNAi_500_.

All plasmid constructions were made using *Escherichia coli* Top 10F’ (Invitrogen) as recipient. The plasmidic DNA was extracted by using the GeneJET Plasmid Miniprep Kit (Thermo Scientific, Carlsbad, CA, USA). All the constructs were sequenced to confirm that they were correct.

### 2.4. Transfection and Selection of Giardia Trophozoites Containing the Vectors

*Giardia* trophozoites were transfected essentially as previously reported [26]. Trophozoites were grown at 37 °C in TYI-S33 culture medium; the cells were harvested at mid-to-late logarithmic phase by chilling the culture tubes on ice for 10 min and collected by centrifugation (1000× *g*/10 min at 4 °C).

Upon placing a concentrated cell suspension (300 μL containing 1 × 10^7^ cells) in a previously chilled 0.4 cm-gap electroporation cuvette, plasmidic DNA (50 μg) was added, and the mixture was incubated on ice for 5 min. Next, cells were electroporated using the following conditions: 350 V, 1000 μF and 700 Ω in a GenePulser Xcell (Bio-rad, Hercules, CA, USA). After electroporation, the cuvette was placed back on ice for 15 min, and then the cells were transferred to 7 mL of culture medium in a capped glass tube [28].

The electroporated cells were incubated 24 h/37 °C without puromycin; after that, the culture medium of the trophozoites was exchanged with fresh medium containing increasing concentrations of puromycin (30, 60 and 100 μM); exchanges were done after 48, 72 and 96 h, respectively, as previously recommended [29,30]. The cells were then preserved in dimethyl sulfoxide (DMSO) and stored in liquid nitrogen.

### 2.5. Confocal Microscopy

All transformant cells obtained with the p*TubGdh*_eGFP-RNAi vector were cultured overnight in 12-well microplates (Corning, Corning, NY, USA) with 1 mL of TYI-S-33 medium, containing a coverslip for the addition of the trophozoites. Next, the cells on the coverslip were fixed with cold methanol for 3 min, washed twice with phosphate buffer (pH 7.4) and mounted with Fluoroshield (Sigma-Aldrich, St. Louis, MO, USA). The samples were analyzed and photographed with a confocal microscope (Olympus FV1000).

### 2.6. RNA Extraction: Total RNA and Small RNA

Total RNA of the WB strain and transformant cells was extracted at different growth times by using the TRIZOL reagent method (Invitrogen^TM^). RNA was treated with DNAse I (Thermo Scientific) to prevent DNA contamination of the samples; RNA concentration and purity were then determined using a μDropTM plate (Thermo Scientific).

The extraction of small RNAs was performed with the mirPremier^®^ microRNA Isolation Kit (Sigma-Aldrich), following the instructions of the manufacturer’s protocol. The small RNA obtained was electrophoresed in 3 % (*w*/*v*) agarose gels and visualized with GelRed (Nucleic Acid Gel, Biotium; Hayward, CA, USA) on a MultiDoc-It (UVP; Upland, CA, USA).

In addition, RNAs with sizes ≈ 100 bp were cut from acrylamide gels and purified using the aforementioned kit, following the protocols of Malone et al. [31] and Nielsen [32], with some modifications.

### 2.7. cDNA Synthesis and Reverse Transcription PCR (RT-PCR)

For RT-PCR, 100 ng of purified *Giardia* RNA were mixed with 0.5 μg of oligo-dT (Thermo Scientific) and 15 μL of DEPC-Treated Water (Invitrogen); the mixture was incubated at 70 °C/5 min to denature the RNA. The cDNA was synthesized using an oligo(dT)_18_ primer and the reverse transcriptase *Revertaid* (Thermo Scientific). Amplification of gene fragments from cDNA was performed using specific primers and a high fidelity DNA polymerase in a final volume of 25 μL, with the following PCR conditions: 5 min at 94 °C; 30 cycles of 30 s at 94 °C and 60 s at 62 °C; and 7 min at 72 °C. Amplification products were analyzed by electrophoresis in 1.5% agarose gels and stained with GelRed (Nucleid Acid Gel, Biotium).

### 2.8. Quantitative PCR Analysis of Gene Expression

Gene expression of *NADHox* was quantified by RT-qPCR using a StepOne^TM^ Real-Time PCR System (Applied Biosystems, Foster City, CA, USA) and the Fast SYBR Green Master Mix kit (Applied Biosystems). Oligonucleotides were designed using the *Giardia NADHox* gene sequence in GenBank (id: XP001707974.1), outside of the region chosen to generate the dsRNAs. GiardiaDB: The Giardia Genomics Resource [25] was used for the designing. The housekeeping actin (*Act*) gene (GenBank id: XM_001704601) was used as a control for constant expression [25]. Quantification of the expression of mRNA for both *NADHox* and *Act* genes was determined by the ΔΔCt method (Ct: threshold cycle), accordingly with the following equations: Δ*C*_t._ = *C*_t._ (target) − *C*_t._ (normalized) and ΔΔ*C*_t._ = Δ*C*_t._ (experimental) − Δ*C*_t._ (control); the comparative expression level was given by the value of 2^− ΔΔ^*^C^*^t.^ [27,33].

### 2.9. Small RNA Specific Stem-Loop RT-qPCR

For the detection and quantification of possible siRNA formed from the synthesis of specific fragments of dsRNA, we used the stem-loop technique [34,35]. This methodology consists of two steps: reverse transcription (RT) and real-time PCR. First, the stem-loop probe, stem-loop–NADHox, was used to capture the possible siRNA generated from the 200 bp dsRNA and perform reverse transcription with the Revertaid reverse transcriptase (Thermo Scientific). Then, the RT product was quantified by RT-qPCR using SYBR Green and the primers siRNA NADHox Fw and UniLoop Rv. The sequences of the specific Stem-loop NADHox and UniLoop primers were designed using the Universal Probe Library probe #21 (UPL21) which was obtained with the software miRNA Primer Design Tool [34]. Quantification of the expression of siRNA and snoRNA was performed with the ΔΔCt method; the actin (*Act*) gene was used as control for constant expression.

### 2.10. Assay of NADHox Enzyme Activity

The enzyme activity of NADHox from wild type (WT) and transformant trophozoites was assayed with spectrophotometry. Around of 1 × 10^7^ cells were centrifuged at 1800× *g*/10 min/4 °C, the supernatant was discarded and the pellet was resuspended in 50 mM Tris pH 8; this procedure was repeated three times. After that, 1 × 10^7^ cells were sonicated for 6 cycles of 15 s at 4 °C in the same buffer supplemented with a protease inhibitor cocktail (Sigma-Aldrich). The sample was centrifuged at 12,300× *g*/20 min at 4 °C and the supernatant was collected. Then, a 4 μL sample was added to a 1 mL of reaction mixture containing 50 mM Tris pH 8 and 0.2 mM NADH. The activity was measured at 25 °C following the decay in absorbance at 340 nm during the first 3 reading min; the data are reported as nmol of NADH oxidized × min^−1^ × 1 × 10^−7^ cells [36].

### 2.11. Statistical Analysis

All results were expressed as means ± standard deviations (SD). A first analysis was performed via one-way ANOVA in order to identify overall differences in each of the studied times. Where ANOVA showed significant differences, comparisons between the transformants and WT cells were performed. All statistical analyses were carried out with the statistical software GraphPad Prism version 5 (GraphPad Software Inc., La Jolla, CA, USA). *p* value < 0.05 was considered statistically significant.

## 3. Results

The NADHox enzyme of *G. lamblia* is thought to act reducing intracellular oxygen to water in order to protect oxygen-labile enzymes such as pyruvate-ferredoxin oxidoreductase [24]. Considering that the *NADHox* gene plays a fundamental role in the redox metabolism of *Giardia*, we decided to study the effect of silencing this gene through an RNAi approach.

### 3.1. Construction of Vectors for dsRNA Synthesis in Giardia lamblia

For the silencing of the *NADHox* gene, four plasmids were constructed based on the p*TubGdh*-RNAi vector and four more based on the p*TubGdh*-eGFP-RNAi vector, containing DNA fragments from this gene ranging from 200 to 500 bp. We used this broad interval of sizes because there are no references about the most efficient dsRNA size for RNAi in an early eukaryotic organism such as *Giardia*. The selected *NADHox* gene region for amplifying the fragments spans from +500 to +1203 bp with respect to the ATG start codon. First, a 704 bp fragment was amplified by PCR with primers containing an NcoI site at the 5′-end (Table 1). In this fragment an NcoI site is present which generates two fragments of approximately 200 and 500 bp (Figure 1B). The rest of the fragments were obtained by PCR using the primers mentioned in Table 1. The constructed plasmids were accordingly named p*TubGdh*-RNAi_200_ through p*TubGdh*-RNAi_500_ and p*TubGdh*-eGFP-RNAi_200_ through p*TubGdh*-eGFP-RNAi_500_, and include the *α2*-*Tub*::*gdh* cassette, with an NcoI cloning site that allows the insertion of the DNA fragments which will be transcribed from both strands to form a dsRNA (Figure 1C). Both promoter regions induce strong constitutive transcription [37,38] to favor the constant generation of dsRNAs, which would induce the enzymatic system of the *G*. *lamblia* RNAi pathway, causing the silencing of the *NADHox* gene (Figure 1C). All the constructed plasmids were digested with the restriction enzyme NcoI to confirm the presence of the inserted fragment (Figure 1D).

### 3.2. Analysis of the Insertion of the Silencing Vectors into Giardia Trophozoites

After transformation of the trophozoites with the p*TubGdh*-RNAi and p*TubGdh*-eGFP-RNAi-derived plasmids, the following tests were conducted to check whether they were integrated into the genome of *G*. *lamblia*.

**Figure 2:**
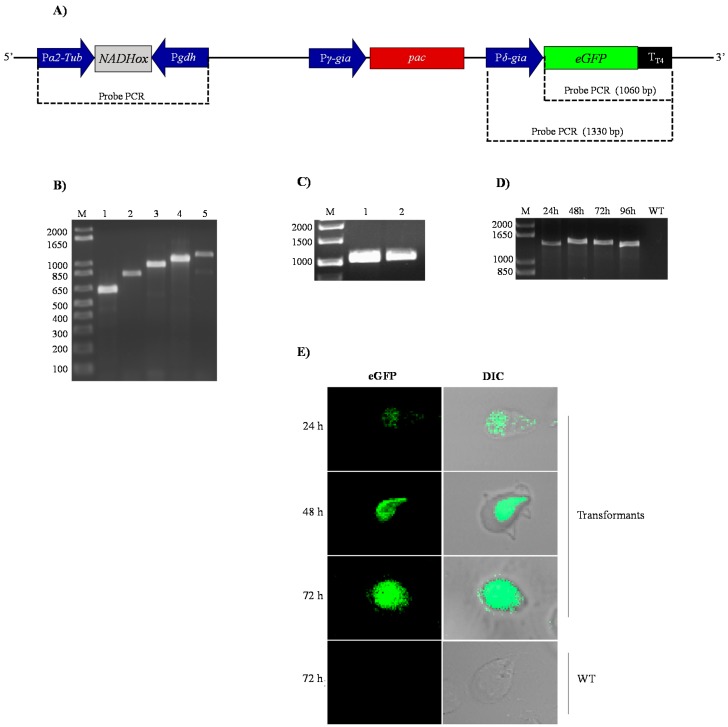
Analysis of *Giardia* trophozoites transformed with the p*TubGdh*_eGFP-RNAi derived plasmids for the presence of elements of the vector and *eGFP* gene expression ability. (**A**) Linearized p*TubGdh*_eGFP-RNAi vector showing the *α2-Tub*::*gdh* and the *δ-Gia*::eGFP cassettes for dsRNA synthesis and *eGFP* gene expression, respectively, and the position of primers used for PCR with the expected amplicon sizes. (**B**) Electrophoresis gel of the PCR amplification products obtained from transformants with different fragments of the *NADHox* gene inserted at the *α2*-*Tub*::*gdh* cassette; lane 1: transformant with the empty *α2-Tub*::*gdh* cassette (650 bp); lanes 2 to 5: transformants with the *α2-Tub*::*gdh* cassette containing *NADHox* gene fragments of 200, 300, 400 and 500 bp, respectively; the primers used were *α2-Tub* Fw and *gdh* Rv (Table 1). (**C**) Lanes 1 and 2 show the PCR amplification product of 1060 bp corresponding to the *eGFP* gene, using primers *eGFP* Fw and *eGFP* Rv. (**D**) PCR products obtained from the amplification of the entire *δ-Gia*::*eGFP*::T_T4_ cassette, using as template DNA obtained at 24, 48, 72 and 96 h of cultivation of transformants; WT corresponds to the PCR performed with the wild type strain; M is the molecular weight marker. The primers used were *δ-Gia* Fw and *eGFP* Rv. (**E**) Detection by fluorescence confocal microscopy of the eGFP protein in *G*. *lamblia* transfected with plasmid p*TubGdh*_eGFP-RNAi_200_ (containing the 200 bp fragment of the *NADHox* gene). The image reveals the presence at different times of cultivation of the eGFP protein in the cytoplasm of cells, and the same field analyzed by differential inference contrast, indicated as DIC.

First, the transformant *G*. *lamblia* trophozoites were selected by incubation with rising concentrations of puromycin (see Material and Methods); the surviving cells were considered as transformants. After that, genomic DNA from the transformants was used as template to amplify the *α2-Tub*::*gdh* cassette containing the different *NADHox* fragments. The results confirmed that the transformant cells contained the p*TubGdh*-RNAi or p*TubGdh*-eGFP-RNAi vectors with the empty cassette (650 bp) or with the cassette harbouring the different *NADHox* fragments inserted into their genome (Figure 2B). All amplified fragments were sequenced to discard possible errors. We used this broad interval of sizes because there are no references about the most efficient dsRNA size for RNA interference in an early eukaryotic organism such as *Giardia*. The selected *NADHox* gene region for amplifying the fragments spans from +500 to +1203 bp with respect to the ATG start codon.

Second, we characterized the cassette that contains the green fluorescent protein (*δ-Gia*::*eGFP*) in the transformants with the vector p*TubGdh*-eGFP-RNAi (Figure 2A). For this aim, genomic DNA of transformant cells was used as the template to amplify by PCR the *eGFP* gene, which is not naturally present in the genome of *Giardia*. An amplified fragment of 1060 bp was obtained, whose sequence corresponded to that of the *eGFP* gene, thus confirming the integration of the vector into the *Giardia* genome (Figure 2C). Similar PCR tests were also used by Gourguechon and Cande [39] to confirm integration of plasmids into the genome of *Giardia*. Another PCR assay was performed with primers designed to amplify the entire *δ-Gia*::eGFP::T_T4_ cassette (Figure 2D); an amplified DNA band of about 1330 bp was obtained in trophozoites cultured for 24 to 96 h. As expected, no amplified band was obtained from trophozoites of the WT strain.

Expression of the eGFP protein was analyzed by confocal microscopy. The fluorescence signal was distributed homogeneously in the cytoplasm of transformant cells (Figure 2E). It is also noteworthy that no changes in the phenotype of trophozoites were appreciated due to the presence of the vector or by silencing of the *NADHox* gene, as observed in the trophozoites containing the 200 bp fragment of the *NADHox* gene. These results indicate that the transformation of trophozoites by electroporation was effective and that, according to the performed tests, the plasmid was stably integrated into *Giardia* cells (Figure 2D) and transcription of the *eGFP* gene occurs from the beginning of trophozoite growth until, at least, 72 h (Figure 2E).

### 3.3. Effect of the Different Sizes of dsRNA on the NADHox Gene Silencing Efficiency

The DNA fragments inserted at the *α2-Tub*::*gdh* cassette are transcribed from both sides by constitutive strong promoters, and thus dsRNA from these fragments is expected to form constitutively. The dsRNA will be targets for Dicer processing to generate siRNA, thus causing specific silencing of the *NADHox* gene expression. Nevertheless, differences in strength between the two promoters are likely to exist, which would cause an asymmetry in the amount of synthesized RNA between strands, and therefore single-stranded RNA (ssRNA) molecules are also expected to form corresponding to the strand transcribed from the stronger promoter. ssRNA may be processed to form miRNA-like molecules, as will be described below.

We evaluated the ability of *NADHox* gene fragments ranging in size from 200 to 500 bp to knock down *NADHox* gene expression through dsRNA synthesized by sense and antisense transcription at the *α2-Tub*::*gdh* cassette of the p*TubGdh*_eGFP-RNAi vector. The transformant cells carrying the different *NADHox* gene fragments were accordingly named N200 through N500. *NADHox* expression was determined by RT-qPCR, using WT trophozoites as control. For this first quantification, RNA was obtained from cells grown for approximately 50 h, before reaching monolayer. This initial screening showed that transformants N200 showed the highest attenuation efficiency (31%), whereas in transformants N300 and N400 efficiencies were close to 15% and in N500 no attenuation was observed (Table 2). Considering these results, we selected transformant cells showing higher (N200) and lower (N400) ability to interfere with *NADHox* gene expression in order to determine the effect upon cellular growth as well as enzyme activity of NADHox.

### 3.4. Gene Silencing of NADHox Delays Cellular Growth and Decreases the Enzymatic Activity

**Figure 3:**
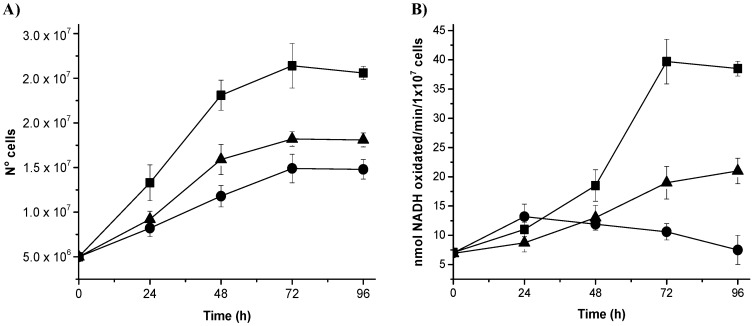
Kinetics of cell growth (**A**) and NADHox enzyme activity (**B**) from the wild type (WT) strain (■) and N200 (●) and N400 (▲) transformants. The figures represent the average of three independent experiments. However, each of these three experiments were performed in triplicate, so the standard deviation is the reflection of 9 replicates at each time. For all experiments, the standard deviation was less than 10%.

We analyzed whether the decrease of mRNA from *NADHox* correlated with changes in the growth rate of *Giardia* by comparing the growth rates of transformant trophozoites expressing dsRNA from *NADHox* with those of the WT strain. Transformants N200 and N400 showed a delayed growth after 24 h of cultivation; the effect was more evident after 72 h (Figure 3A). The growth of N200 was more affected than that of N400 cells, which correlates with the stronger silencing of *NADHox* mRNA expression observed in the former with respect to N400 (Table 2). To confirm the effect of *NADHox* silencing on the expression of the protein, we determined its enzymatic activity in extracts from the three trophozoite strains during different periods of cultivation. Significant differences in enzyme activity were observed between the three strains after 48 h of cultivation; at 96 h the NADHox activity in N400 and N200 strains was, respectively, 2-fold and 5-fold lower than that in the WT strain (Figure 3B).

Taken together, these data confirm that the vector constructed to generate NADHox dsRNA is capable of attenuating NADHox expression and consequently reduce NADHox enzymatic activity, which in turn has implications on the growth of transformant trophozoites.

### 3.5. Kinetics of Attenuation of NADHox Gene Expression in the Transformants N200 and N400

**Figure 4:**
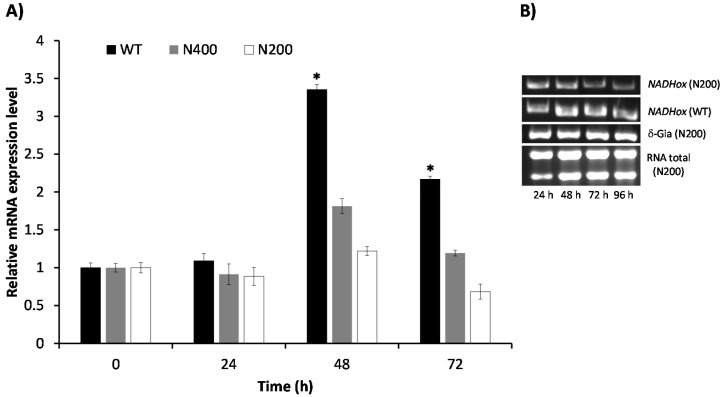
Quantification of *NADHox* mRNA in the wild type (WT) and transformants with a silenced *NADHox*. (**A**) Kinetics of mRNA expression of the *NADHox* gene from the WT strain (black bars) and the N200 (white bars) and N400 (gray bars) transformants. mRNA quantitation was performed by RT-qPCR (see Materials and Methods). The primers used for analysis of the *NADHox* and *Act* transcripts were *qNADH* and *qAct*, respectively (Table 1), using the actin gene (*Act*) (GenBank id: XM_001704601) as housekeeping gene control. The figures represent the average of three independent experiments with three replicates each experiment and in real time each replicate was quantified 5 times. The results at 48 and 72 h of cultivation showed significant differences between strains (* *p* < 0.01 with one-way ANOVA). (**B**) The transcript levels of the *NADHox* gene at different times was also determined by semiquantitative RT-PCR for the WT strain and N200 transformants; the *δ-Gia* gene was used as housekeeping control.

The effect of attenuating the expression of the *NADHox* gene on the decay of enzyme activity was observed only after 48 h of cultivation of the transformant cells. To explore if the delay in the reduction of NADHox enzymatic activity was due to mRNA degradation beginning after 48 h, we determined the levels of the *NADHox* transcript at different cultivation times in both N200 and N400 strains by RT-qPCR. In the WT strain, the *NADHox* mRNA levels showed a transient 3.5-fold increase at 48 h of cultivation, followed by a decrease at 72 h, when mRNA levels were 2-fold the levels at 0–24 h (Figure 4). These data correlate well with the results shown in Figure 3B, where the highest translation levels of this mRNA are inferred to be at around 60 h of cultivation from the pattern of NADHox enzyme activity, which was highest at 72 h and did not change significantly at 96 h. Hence, the data indicate that retardation in the effect of silencing on enzyme activity may be due to the fact that the *NADHox* gene exhibits important transcription levels only at around 48 h; before this time the expression of the *NADHox* gene is probably insufficient to observe significant effects on NADHox enzyme activity through attenuation of *NADHox* expression.

The N400 transformants showed an inconspicuous increase of the transcript levels at 48 h of cultivation (1.8-fold), whereas at 72 h the level of transcript was similar to that at 0–24 h. In contrast, the N200 transformants showed only a minor increase at 48 h (1.2-fold), and the lowest *NADHox* mRNA levels were observed at 72 h.

Since the N200 transformant showed a better response to interference RNA than N400, we decided to corroborate its behavior following the decay of the *NADHox* transcript by semiquantitative RT-PCR and comparing the data with those of the WT strain (Figure 4B). The results confirmed that, indeed, the amplification bands of the *NADHox* transcript from the N200 strain gradually decreased in intensity during the time course of the experiment, whereas in the WT strain the intensity underwent a transient increase similar to the previously observed in Figure 4A. The expression of the housekeeping reference gene *δ-Gia* remained constant throughout the cultivation time.

### 3.6. Detection of Small Interfering RNA by Stem-Loop RT-PCR

**Figure 5:**
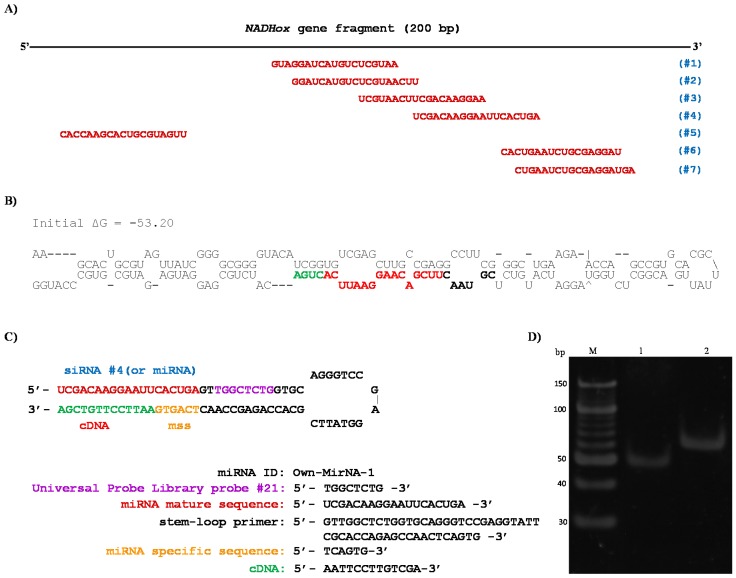
In silico analysis of the 200 bp fragment sequence from the *NADHox* gene for the formation of small RNAs and their detection by stem loop RT-PCR. (**A**) In silico prediction of siRNAs from the 200 bp fragment, using the online tool (bioinfo.clontech.com/rnaidesigner/sirnaSequenceDesignInit.do. (**B**) Predicted secondary structure of the ssRNA (200 bases) obtained with the MFold Web Server, indicating the position of a putative miRNA. (**C**) Structure of the Stem-loop NADHox primer obtained with the Universal Probe Library probe #21 hybridizing to the target siRNA. (**D**) RT-PCR amplified fragments of the stem-loop probe with the captured siRNA run on a 16% polyacrylamide gel: Lane 1, siRNA amplification with a size of ~53 bp, using primers siRNA NADHox Fw and UniLoop; Lane 2, amplification of the stem-loop region (stem-loop NADHox primer) with the specific siRNA #4 (or miRNA) showing the expected size of 63 bp; M, O’RangeRuler 10 bp DNA Ladder (Thermo Scientific).

In order to confirm that silencing of *NADHox* gene expression was caused by siRNAs derived from the processing of dsRNA, we used the stem-loop RT-qPCR technique to identify and quantify this type of small RNAs [34,35,40]. The strongest silencing was observed with the 200 bp *NADHox* fragment; for this reason, an in silico analysis was performed on this sequence using an online tool (bioinfo.clontech.com/rnaidesigner/sirnaSequenceDesignInit.do) to find putative siRNAs that could be generated. The program predicted seven 19-base sequences as potential siRNAs (Figure 5A). The 200 bp fragment is the only region containing these seven possible siRNA sequences due to its position in the gene (Figure 1B). In the rest of the fragments of 300, 400 and 500 bp, putative siRNAs were also found in silico, however the sizes of these fragments are not adequate for Dcr processing since this RNase shows better activity with shorter dsRNA, as previously reported by MacRae et al. [20,21], which is in accordance with the stronger attenuation of *NADHox* observed in transformants N200 (Table 2).

As indicated above, single strand RNAs may also be produced by asymmetric levels of transcription at the *α2-Tub*::*gdh* cassette. Therefore, we performed in parallel an analysis for prediction of possible secondary structures in the ssRNA of the 200 bp sequence, using the MFold Web Server [41]. For the analysis we used the strand transcribed from the *α2-Tub* promoter, since it has been previously reported as a very strong promoter of constitutive expression in *G*. *lamblia* [38]. Interestingly, the sequence formed a hairpin loop structure (Figure 5B) qualified as a suitable substrate for Dcr activity [42]. This structure resembles those described for some small nucleolar RNAs (snoRNAs) from *G*. *lamblia*, such as GlsRNA16 and GlsRNA17 [16], including the presence of a D box (shown in green in Figure 5B) characteristic of this type of structures and recognized by Dcr [20,21]. In addition, in the hairpin loop structure was located a 21 bp sequence that was identified as a possible miRNA (shown in bold, black and red colors in Figure 5B). Coincidentally, this putative miRNA is similar in sequence to the predicted siRNAs #3 and #4 (Figure 5A). When ssRNA sequences from the rest of the fragments used for silencing (Figure 1B) were analyzed with the same tool no secondary structures were found with the features of the above mentioned for the 200 bp fragment (not shown).

In view of this, we consider that the silencing of the *NADHox* gene in N200 transformants may be due to the formation of both siRNAs and miRNAs. To verify that these small RNAs are generated we used the stem-loop RT-qPCR method as previously described [34,35]. The sequences and secondary structure of the stem-loop design are shown in Figure 5C; the stem-loop probe was specifically designed to capture the predicted siRNA #4 and miRNA.

With this purpose, we purified small RNA of less than 100 bp in size from previously obtained total RNA, as reported by Marcial et al. (2016) [35], to use these fractions as a template in the RT-PCR tests for the isolation of siRNA (or miRNA). According to the design of the primers and to the procedure of the stem-loop technique, when PCR amplification was performed, two products were observed (Figure 5D). A 53 bp fragment was obtained in the PCR reaction using primers siRNA NADHox Fw and UniLoop (Table 1), which corresponds to the amplification of the siRNA #4 and a region of the stem-loop including the UniLoop primer (Figure 5D, lane 1). Another fragment of 63 bp was amplified which corresponds to the entire stem-loop region (Stem-loop NADHox primer) and the specific siRNA (Figure 5D, lane 2). When purified small RNA of less than 100 bp from the WT was used as template no amplified fragments were obtained. These results indicate that a 200 bp dsRNA is synthesized from the vector p*TubGdh*_eGFP-RNAi_200_ and is then processed to generate siRNAs which silence expression of the *NADHox* gene, not excluding the possibility of generation of a miRNA-like molecule from the 200-base ssRNA shown in Figure 5B.

### 3.7. Quantification of siRNA in the N200 Transformant

**Figure 6:**
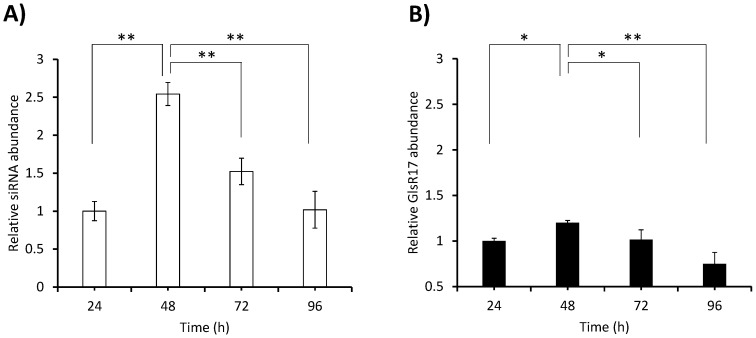
Quantification by RT-qPCR of siRNA (**A**) and snoRNA (GlsR17) (**B**) levels in trophozoites of the N200 transformant grown in TYI-S-33 medium. The error bars indicate standard deviation (SD) from five replicates. Statistical analysis by ANOVA and Tukey–Kramer test showed significant differences between 48 and 96 h of cultivation. A * *p* value < 0.05 and ** *p* value < 0.01 were considered statistically significant.

Finally, the expression of the siRNA was quantified in trophozoites grown in TYI-S-33 medium for 96 h with samples taken every 24 h. The analysis was performed by RT-qPCR according to the conditions previously established by Marcial et al. [27]. As mentioned in the previous section, no RT-PCR amplification product was obtained with the stem-loop NADH primer using small RNA (~100 bp) from the WT as template, and therefore no quantification by RT-qPCR was performed in this strain. In the N200 transformant, important differences in abundance were observed between time points (Figure 6A). Considering that the snoRNA named GlsR17 is constitutively expressed [35], we considered it pertinent to quantify its expression over time and compare it with that of the siRNA generated in the same transformant (N200). The highest abundance of the siRNA was observed at 48 h (2.5-fold with respect to the amount at 24 h) and from this point the abundance decreased steadily until at 96 h it was at the same level as at 24 h. Meanwhile the expression of GlsR17 followed a similar trend during the cultivation time, although differences between time points were much less sharp than in the case of the siRNA (Figure 6B). Comparing the abundance of the siRNA in Figure 6A with the abundance of the *NADHox* mRNA in strain N200 with respect to the WT at different times (Figure 4A), a correlation pattern can be observed. The decrease of mRNA in N200 with respect to the WT at 48 h may be explained by the silencing effect caused by the high levels of siRNA at that time; this situation is maintained at 72 h when still high levels of siRNA are present. In contrast, at 24 h, when siRNA abundance is low, the amount of mRNA is just slightly, and non-significantly, lower in N200 with respect to the WT.

## 4. Discussion

In this work, we designed a molecular strategy to test genes encoding potential target proteins for drug design in the parasite *G*. *lamblia*. The strategy is based on the generation of dsRNA to silence expression of a selected gene by the RNAi pathway. Proteins characteristic of the RNAi pathway have been shown to be present and functional in this parasite [11,14,16,21,43], and it has been suggested that expression of some *Giardia* proteins, such as those of the VSP family that serve to evade the attack from the host immune system, are regulated by an RNAi-like mechanism [13,14,15,44]. Along this line, our data show that, indeed, it is possible to induce the RNAi machinery of *Giardia* to specifically attenuate the expression of selected genes. This was possible through the construction of vectors containing a cassette that allows the synthesis of dsRNA fragments from regions of the selected gene. Some studies have shown the presence of dsRNA or antisense RNA from endogenous (*α-Tub*, *RdRP*, *VSP*) or exogenous (*eGFP*) genes, which inhibit expression of these proteins; this is indicative of a gene-specific silencing mechanism in *G*. *lamblia* [15,45].

We tested the ability of the constructed vectors to silence expression of the *NADHox* gene. Reduction of *NADHox* transcript levels in the cells transformed with the silencing vector was maintained for the whole time of the experiment, i.e., 96 h, and this caused considerable effects on the growth of trophozoites and NADHox enzymatic activity.

Our method yielded a maximum of 31% inhibition of *NADHox* expression. Comparable results were also obtained by others using different molecular strategies; for example, a giardiavirus-based ribozyme approach resulted in a knockdown of 30% in the expression of the diaphorase gene [46], whereas for the pyrophosphate-dependent pyruvate phosphate dikinase gene, the attenuation was 20% [47]. Using an antisense RNA method, a 47% knockdown was obtained for the phosphatase 2A gene [48]. These data indicate that our system of interference through dsRNA synthesis is within the expected ranges of attenuation yields for *Giardia*.

When designing an RNAi strategy for gene silencing, it is important to find the DNA fragment sizes that will give better attenuation efficiencies. Our results suggest that the highest efficiency for RNAi-mediated gene silencing in *Giardia* is obtained with small dsRNA around 200 bp. Fragments with higher sizes resulted in substantially lower attenuation efficiency. These results contrast with previous reports in humans and fungi such as *Ophiostoma*, in which the highest yields were achieved using fragments around 500 bp [49,50]. Similarly to the organisms just mentioned, better results using fragments of larger sizes were also observed in an RNA interference approach in *Trypanosoma brucei* [51,52,53,54]. Such differences in the selectivity of the dsRNA size for gene silencing may be due to the intrinsic properties of the protein complexes involved in the RNAi mechanism, for instance, the Dcr protein [21], and possibly homologs to dsRNA-binding proteins, as has been suggested before [10]. Hence, whereas in the early divergent eukaryote *Giardia*, Dcr apparently shows higher activity with shorter dsRNA, which was demonstrated using Dcr in the presence of 150 bp fragments by an in vitro cleavage assay [20,21], the enzymes of later divergent organisms such as trypanosomatids, fungi or humans have preference for longer dsRNAs. In addition, we cannot exclude the possibility that the higher inhibition of *NADHox* expression observed with the 200 bp fragment was also partially due to the likely generation of miRNA-like molecules from the 200-base ssRNA (Figure 5B), which are unlikely to form from other fragments according to in silico analysis. Both the siRNA derived from dsRNA and the miRNA derived from ssRNA would contribute to the silencing of the *NADHox* gene in the N200 transformants.

The knockdown of *NADHox* gene expression resulted in a significant decrease of enzyme activity, as well as in a reduction of growth in the transformant cells; however, the effect upon this latter parameter was less pronounced. This might be due to the microaerophilic conditions that prevailed during all studies of cell growth; such circumstances could reduce the oxidative stress that hindered the protective contribution of NADHox activity on cell proliferation. Our data correlate with previous reports [48] in which, under anaerobic conditions, the growth of transfectant trophozoites overexpressing *NADHox* showed subtle differences with respect to control cells. Only under aerobic conditions, did the transformant cells show differences increasing their growth with respect to the control cells, which indicated that under oxidative stress this enzyme plays an important role. Following this line, it is illustrative to point out the results obtained when the DT-diaphorase was overexpressed [48]. This enzyme is susceptible to form superoxide anions during its catalysis and promote an oxidative stress condition in *Giardia*, consequently reducing cell growth. This circumstance, however, was again only observed under aerobic conditions in which overexpression of DT-diaphorase decreased the cell number four-fold. Under anaerobic conditions, there were no differences between the wild type and the transfectant cells.

In this context, it has been reported that NADHox exhibits an important antioxidant defense function to protect susceptible enzymes such as pyruvate:ferredoxin oxidoreductase [55]. The effectiveness of NADHox to reduce oxygen to water has been demonstrated before, showing high rates of electron acceptors of 6.0 ± 0.75 mol O_2_ × min^−1^ × 10^−6^ trophozoites [56]. Furthermore, a proteomic and transcriptional study [57] found that NADHox is increased in *Giardia* clones resistant to albendazol, suggesting that this enzyme might also prevent the generation of ROS. Such results correlate with a recent microarray hybridization study of trophozoites under oxidative stress, which showed that genes involved in protection against stress were significantly overexpressed [58].

## 5. Conclusions

In conclusion, this paper describes a useful tool to specifically knockdown gene expression in the parasite *Giardia* through gene silencing by the RNAi pathway. We have constructed vectors that can generate dsRNA from any gene of interest by inserting a gene fragment in a single step. The relevance of this study is the development of a molecular tool for evaluation of genes encoding proteins that may be potential drug targets. The enzymes may belong to several essential metabolic pathways, dealing with energetic, catabolic or anabolic processes, such as glycolysis, a main route for ATP generation in this parasite.

## Figures and Tables

**Table 1:** PCR primers used in this work; the location of the restriction sites in the primer sequences is underlined.

PCR Amplification (Product Size in base pair, bp)	Oligonucleotide Sequences
*gdh* (240)	5′-CCTGGATATCAAGCTTCACGTCGTCGTTCTC-3′ (EcoRV) 5′-ATAACCATGGTTTAAAATCTGGGGCGCCTGT-3′ (NcoI)
*RNAi NADH* (704)	5′-CAATCCATGGTCGAAACGTTTTCTCACG-3′(NcoI) 5′-ATATCCATGGTACCTATCCTCATCTGT-3′(NcoI)
*NADH* (411)	5′-GTCACCATGGGCTATTCTTTGTATCGGCTTC-3′ (NcoI) 5′-AGTACCATGGAGGCCTGTCCGTGTCATTAAT-3′ (NcoI)
*NADH* (309)	5′-ATATCCATGGTACCTATCCTCATCTGT-3′(Nco*I*) 5′-AGTACCATGGAGGCCTGTCCGTGTCATTAAT-3′(NcoI)
*eGFP* (1068)	5′-TTAATCTAGAAAGGAGGTGATCATATG-3′(XbaI) 5′-TAATCTGCAGAGCTCTTTATTATTTTTAAG-3′(PstI,SacI)
*δ-Gia* (240)	5′-ATAAGCGGCCGCTCGATCTTTCCTTGTTG-3′ (NotI) 5′-AGCGTCTAGATTTTTTCTGGCGCGAAAAGC-3′ (XbaI)
*qAct* (60)	5′-TTGCCGTACCTGCCTTCTAT-3′ 5′-GCCCGGAACTGTAGAGAGC-3′
*qNADH* (98)	5′-GCACCATATGGCTTCAACGG-3′ 5′-CAGGCCTGTCCGTGTCATTA-3′
*qδ-Gia* (70)	5′-AGGACGACCAGGAGGAGAA-3′ 5′-ACGGGTAAAGGCACAATTC-3′
Stem-loop NADHox	5′-GTTGGCTCTGGTGCAGGGTCCGAGGTATTCGCACCAGAGCCAACTCAGTG-3′
siRNA NADHox Fw	5′-GTGGGGTCGACAAGGAATT-3′
UniLoop-Rv	5′-GTGCAGGGTCCGAGGT-3′
pJET1.2	5′-CGACTCACTATAGGGAGAGCGGC-3′ 5′-AAGAACATCGATTTTCCATGGCAG-3′

**Table 2:** Comparative content of *NADHox* mRNA in the transformants synthesizing dsRNA from 200 to 500 bp with respect to the WT strain, as measured by RT-qPCR.

Transformants	Mean mRNA Content (±SD) ^2^	Percent mRNA Content Relative to WT (%)
WT ^1^	1 (±0.041)	100
N200	0.697 (±0.015)	69.7
N300	0.885 (±0.079)	88.5
N400	0.837(±0.021)	83.5
N500	1.028 (±0.089)	100

^1^ Wild type (WT) is considered to have a mRNA content value of 1.0, and the mRNA content of the transformants is relative to this. ^2^ Values are the result of three replicate experiments, using *Act* as housekeeping reference gene for normalization.
